# Phytotoxic and Eustress Effects of Metal Oxide Nanoparticles (CuO, Mn_x_O_x_, and ZnO NPs) on Plants

**DOI:** 10.3390/plants15091353

**Published:** 2026-04-28

**Authors:** Elena I. Strekalovskaya, Alla I. Perfileva, Konstantin V. Krutovsky

**Affiliations:** 1 Laboratory of Environmental Biotechnology, A.E. Favorsky Irkutsk Institute of Chemistry, Siberian Branch of the Russian Academy of Sciences, 664033 Irkutsk, Russia; ivanova.iem@gmail.com; 2 Laboratory of Plant-Microbe Interactions, Siberian Institute of Plant Physiology and Biochemistry, Siberia Branch of the Russian Academy of Sciences, 664033 Irkutsk, Russia; alla.light@mail.ru; 3 Department of Forest Genetics and Forest Tree Breeding, Georg-August University of Göttingen, Büsgeweg 2, 37077 Göttingen, Germany; 4 Laboratory of Population Genetics, N.I. Vavilov Institute of General Genetics, Russian Academy of Sciences, Gubkin Str. 3, 119333 Moscow, Russia; 5 Genome Research and Education Center, Laboratory of Forest Genomics, Department of Genomics and Bioinformatics, Institute of Fundamental Biology and Biotechnology, Siberian Federal University, 660036 Krasnoyarsk, Russia; 6 Scientific and Methodological Center, G.F. Morozov Voronezh State University of Forestry and Technologies, Timiryazeva Str. 8, 394036 Voronezh, Russia

**Keywords:** reactive oxygen species, antioxidant enzymes, biometric parameters, dose-dependent effect, malondialdehyde, trace elements, nanoparticles, nanocomposites, nanomaterials, oxidative stress, phytotoxicity, photosynthetic pigments

## Abstract

Nanoparticles (NPs) have great potential for stimulating plant growth and development, reducing the negative impact of various types of stress on plants, and increasing the yield of agriculturally important crops. Metal oxide NPs (MONPs) have been shown to have a significant effect on the physiological and biochemical processes in plants, enhancing plant resilience. Among them, CuO, Mn_x_O_x_, and ZnO NPs are of particular interest because they contain elements essential for plant function. However, widespread use in agrochemistry and plant protection requires a preliminary risk assessment due to their potential phytotoxic effects. Phytotoxicity manifests through the development of oxidative stress, genotoxicity, and transcriptional disruption. A decrease in plant growth and photosynthesis, increased lipid peroxidation (LPO), and the accumulation of toxic NPs in plant tissues were also observed. Among the studied MONPs, CuO and ZnO NPs exhibit the greatest phytotoxic effects. However, the effects of MONPs are dose-dependent. Numerous studies have shown that MONPs can stimulate plant biometric parameters and productivity, as well as influence biochemical processes. MONPs have been shown to influence the functioning of the plant antioxidant system, manifested by modulating the content of reactive oxygen species (ROS), the activity of antioxidant enzymes (AOEs), and the regulation of signaling pathways mediated by ROS and reactive nitrogen species. Furthermore, MONPs influence the accumulation of proline and phenols in plant tissues. MONPs have a pronounced effect on the functioning of the plant photosynthetic apparatus, manifested by changes in pigment content, the activity of photosynthetic enzymes, and the functioning of photosystems. MONPs can improve nutrient absorption, regulate osmotic balance, and activate plant defense mechanisms. ZnO NPs are effective in mitigating salt stress. CuO and Mn_x_O_x_ NPs have shown promise in mitigating biotic stress. Furthermore, these NPs were found to reduce the toxicity of heavy metals to plants. Overall, when used wisely, MONPs hold promise for enhancing the physiological, biochemical, and agronomic performance of crop plants under conditions of global climate change, effectively addressing food security issues.

## 1. Introduction

Due to global climate change, the stress load on plants is increasing due to changing climatic conditions and expansion of phytopathogens [1]. Plant resistance mechanisms to stress factors are complex, as plants simultaneously face a large number of different stressors. Plant resistance to stress is regulated by a number of mechanisms associated with the functioning of signaling pathways and the regulation of gene expression and enzyme activity. A wide range of pesticides and other agrochemicals are actively used to enhance plant resistance. These substances can increase crop yields, but they are not effective against all stress factors and can pollute the environment and negatively impact human and animal health if used in excessive quantities. Nanomaterials (NMs) offer an alternative to such agrochemicals. They can help plants adapt to changing climatic conditions, reducing their negative impact on the environment. In recent years, nanoparticles (NPs) have been considered eustressors—stimuli that activate beneficial adaptive responses and enhance plant’s adaptation [2].

The production of synthetically synthesized NMs is growing exponentially, and their applications are rapidly expanding. Metal oxide NPs (MONPs) have been actively researched in recent decades [3]. Among the MONPs, copper (Cu), manganese (Mn), and zinc (Zn) compounds are vital for plants and are essential micronutrients for the growth and development of all plants [4]. They are required for development, reproduction, and signaling in plant tissues and organs. Cu, Mn, and Zn also act as cofactors for many plant enzymes [5]. A deficiency of these elements can cause serious diseases and low plant productivity, but at high doses, these elements have a toxic effect [6]. In soils, these chemical elements can be present in various forms: as free ions and in complexes with organic matter. These chemical elements are found in the colloidal fraction of soil in combination with humic substances, bind with other metal compounds, and can form insoluble complexes. The bioavailability of ionic forms of Cu, Mn, and Zn compounds is controlled by adsorption–desorption processes, liquid-to-solid solubility ratios, and depends on pH [7], the chemical and organomineral composition of the soil, root exudates, and substances synthesized by the rhizosphere microbiota. Unlike ionic forms, MONPs actively interact with plants, influencing plant-microbe interactions. At the biochemical level, this manifests itself through the modulation of oxidative stress in plant tissues, activation of signaling pathways associated with stress responses, and the induction of systemic acquired resistance [3]. Therefore, MONPs are considered promising agents for plant health improvement against phytopathogens as an alternative to conventional pesticides. However, the benefits of MONPs are observed only within a narrow concentration range. Exceeding certain doses can lead to phytotoxicity associated with NP accumulation in tissues. To properly use MONPs for plant health improvement, it is necessary to consider the application methods, their concentrations, and the characteristics of the plant being treated.

Published data on the effects of Cu, Mn, and Zn oxide NPs on plants are numerous and controversial. This is due to the different experimental conditions (various NP concentrations, different plant treatment methods, and different growing seasons), as well as the different study subjects, both plants and nanomaterials. All of these published data require further systematization and analysis to more clearly understand the possible mechanisms by which MONPs influence plants, as well as the potential for their use as growth promoters, sources of minerals, and agents for enhancing plant resistance to phytopathogens.

Thus, this review examines the negative and positive aspects of the influence of MONPs (CuO, Mn_x_O_x_, and ZnO NPs) on the physiological and biochemical parameters of plants, their role in protecting plants from stress, as well as phytotoxicity.

## 2. Applications of MONPs

NMs are materials in which at least one of the dimensions of their structural elements is in the nanometer range (1–100 nm). Nanotechnology, the field of science and engineering devoted to the study and application of these NMs, is rapidly and continuously evolving. At this scale, the properties of materials undergo significant changes. Their characteristics, such as solubility, reactivity, spectroscopy, electrical and magnetic properties, and membrane transport, typically differ from those of the same materials at larger scales. These unique properties of NMs open up opportunities for a wide range of applications and promise revolutionary advances in various fields of science and engineering [8]. Potential applications of artificial NMs include advanced materials, display technologies, electronics, nutrition, cosmetics, drug development, and many other applications. Despite such a broad range of applications, the use of NMs can be associated with risks. The small size of NMs may have serious toxicological consequences, since they are able to penetrate into various eukaryotic and prokaryotic cells [9].

Although there are numerous published studies on the toxicological and environmental characteristics of synthetic NMs [10], the information on the magnitude of engineered NMs being produced and potentially released to the environment is insufficient [11]. When modeling material flows from products into the environment and predicting environmental impacts, production volume is an important input variable [12].

To date, various types of NMs have been synthesized, such as metallic and non-metallic NMs, core–shell NMs, composites, organic NMs, NPs, and MONPs. According to the Nanotechnology Products Database (https://product.statnano.com, accessed on 5 March 2026), more than 11,147 manufactured products in various categories containing NPs and NMs are known, involving 3909 companies from 68 countries. Moreover, the number of products containing NPs is growing weekly [13]. According to the Project on Emerging Nanotechnologies’ Consumer Products Inventory (Nanotech Project, https://www.nanotechproject.tech, accessed on 5 March 2026), global industry produces at least 1600 nanotechnology-based consumer goods spanning numerous product categories (e.g., health and fitness, electronics, automotive, appliances, clothing, cosmetics, food and beverage), with many subcategories of raw materials, components, and industrial applications identified in the inventory. The global nanotechnology market has expanded rapidly in recent years; it was valued at approximately USD 206.7 billion in 2025 and is projected to exceed USD 1.1 trillion by 2034, reflecting the growing industrial application of nanomaterials, nanodevices, and nano-enabled technologies across multiple sectors [14].

**Figure 1:**
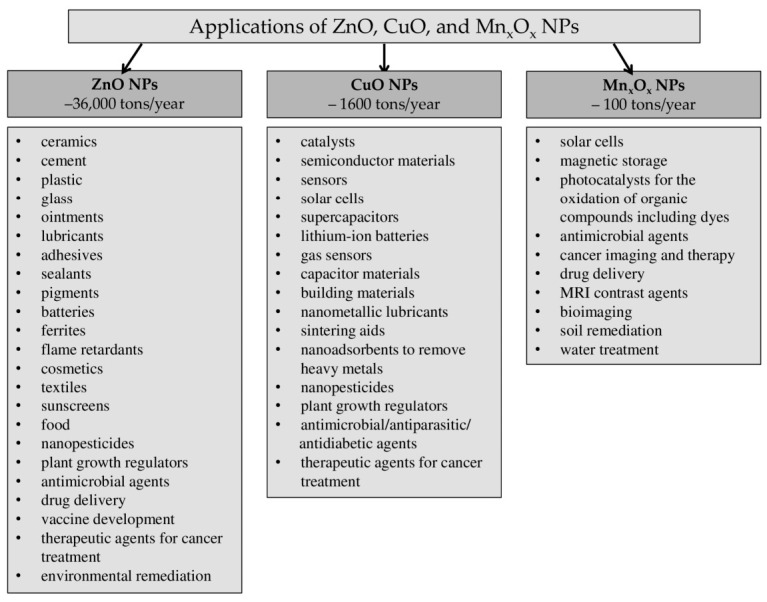
Different practical applications of ZnO, CuO, and Mn_x_O_x_ NPs.

Metallic NPs of Zn, Cu, and Mn in the form of oxides (ZnO, CuO, and Mn_x_O_x_ NPs) are more resistant to external influences, which is why they have gained widespread use. Global production volumes of ZnO NPs range from 550 to 36,000 tons per year (t/y). ZnO NPs are among the top three most popular metallic NPs [15,16]. Global annual production of CuO NPs is estimated at 290–570 t/y. By 2025, production was projected to increase to 1600 t/y [17,18]. The lowest global production volumes (3.5 t/year) are for Mn_x_O_x_ NPs [15]. However, little published data on the production volumes of metallic NPs are available. Furthermore, these data often refer to production capacity rather than actual production volumes, which can vary significantly. Actual data on global NP production are not yet available. Different practical applications of ZnO, CuO, and Mn_x_O_x_ NPs are presented in Figure 1.

In recent years, NPs have begun to play an important role in a wide range of applications. Nanosized ZnO particles have been widely used in various commercial products and additives, including ceramics, cement, plastic, glass, ointments, lubricants, adhesives, sealants, pigments, batteries, ferrites, flame retardants, cosmetics and sunscreens, and in food as a source of Zn [19,20]. Among NMs, Cu NPs are regarded as a potential material for various purposes, such as catalysts, semiconductor materials, sensors, capacitor materials, building materials, nanometallic lubricants, antimicrobial and biocidal agents, and sintering aids [21,22,23,24,25,26,27]. Among the transition metal NPs, Mn NPs have attracted the most attention, especially for applications such as solar cells, magnetic storage, and biological applications including bioimaging [28,29,30].

In recent years, the MONPs have also attracted attention due to their widespread use in agriculture [31,32,33,34,35,36,37]. They act as fertilizers, as they are well absorbed by plants, and MONPs are also used for the mitigation of abiotic stresses, reducing the negative impacts of drought, extreme temperatures, and soil salinity [33,34,35,36,37].

The most popular metallic NPs for the production of nanofertilizers are Zn, Cu, and Mn [38,39,40]. Due to their nanometric size (from 1 to 100 nm), NPs have an increased specific surface area, which contributes to their increased reactivity and interaction with the environment [41,42]. Therefore, for the effective use of NPs in agriculture, it is necessary to understand the mechanisms of their action and key interactions with both the physical and biological environment of the soil, but information in this area is still limited. As microelements, Zn, Cu, and Mn are essential for the proper functioning of plants. They participate in enzymatic processes, photosynthetic activity (chlorophyll formation), protein and carbohydrate metabolism, regulate most redox processes, promote nitrogen assimilation, and enhance the respiration rate and the water-holding and water-absorbing capacity of plants. They also generally influence plant growth processes and the ability to resist adverse environmental conditions (drought, frost, and heat resistance) [35,37,43,44,45]. The potential of NPs in agriculture, due to the increase in crop yield and quality through the interaction of NPs with plants and soil at the molecular level, attracts specialists of various levels [42]. Microelements such as Zn, Cu, and Mn in the form of NPs for use in agrochemistry are studied primarily in the oxide form. When optimal concentrations are exceeded, these microelements, including in nanoform, become potentially hazardous and toxic to plants and soil organisms. Therefore, regular assessment of the phytotoxicity of NPs is of paramount importance for environmental protection [9,10,46,47].

However, despite the demonstrated applications and practical benefits, metallic NPs, due to their unique physicochemical properties, may pose a certain hazard at all stages of production and use, both for human health and for habitats, causing serious socioeconomic consequences [48,49]. Due to unintentional releases of NPs into the environment during their production, processing, and disposal [50], as well as their intentional use as fertilizers, herbicides, and pesticides, direct contact of NPs with the physical and biological environment of the soil is virtually inevitable.

## 3. Routes of MONP Penetration into Plants

NPs can penetrate plant tissues both through the root system and through aboveground organs and tissues (e.g., cuticle, trichomes, stomata, stigmas, and hydathodes), as well as through wounds and root junctions [51]. Plant roots are usually the first to come into contact with NPs released from the soil [52,53]. Adsorption of ZnO NPs was observed on the root surfaces of ryegrass (*Lolium perenne* L.) and buckwheat (*Fagopyrum esculentum* Moench) [54,55]. Enhanced adsorption of ZnO NPs on the root surface likely occurs due to the release of mucigel, a mucilaginous polysaccharide substance coating the root collar. Upon contact with the root, NPs penetrate the cell wall and epidermal membrane, exerting a significant effect on the root epidermis and cortex, penetrating endodermal and vascular tissues [54]. They then enter the vascular bundle (xylem) and migrate to the stele. However, despite the small size of the pores in plant cell walls, NPs are able to penetrate their cell wall, having a diameter of less than 5 nm. NPs are capable of moving in tissues via two pathways: apoplastic and symplastic. Apoplastic transport occurs outside the plasma membrane and is carried out through extracellular spaces, the cell walls of adjacent cells, and xylem vessels [56]. Symplastic transport, in contrast, involves the movement of water and other substances between the cytoplasm of adjacent cells through specialized structures called plasmodesmata and sieve plates [57]. The apoplastic pathway is important for radial movement within plant tissues. It allows NPs to reach the central cylinder of the root and vascular tissue for further upward movement to the above-ground parts [58]. NPs are distributed and transported to the leaves via the xylem. At high concentrations, NPs typically accumulate in the epidermis, cortex, endodermis, cambium, and xylem of plants [59]. NPs accumulate in plants, which serve as a pathway for transmission to herbivores and further to humans through the food chain [60]. Also, NPs can diffuse from the soil into plant seeds and then spread to the root and other plant organs [61,62].

NPs can penetrate plants not only from the soil, but also through air and water. Thus, while in the air, NPs are adsorbed on plant leaves and then absorbed by epidermal cells. NPs that enter the epidermal cells can then be transported to various organs via symplastic pathways or through the apoplast. When applied foliarly, NPs ranging in size from 4 to 100 nm can easily penetrate the protective layer on the surface of leaves, young shoots, and fruits—the cuticle—by destroying the waxy layer [63]. In a study by Cao et al. [64], NPs with a diameter of 43 nm penetrated the leaves of common broad bean (*Vicia faba* L.) only through the stomata, while particles 1 µm in size did not penetrate. Stomatal size varies among plant species. Stomata, which contain two guard cells, are capable of forming a pore 3–12 μm wide and 10–40 μm long when opening for gas exchange and transpiration [65]. Thus, NPs applied to plants by foliar application can penetrate leaves and easily move to other parts of the plant. However, these processes depend on the characteristics of the NPs themselves. For example, it was shown in *Brassica chinensis* L. that the spread of ZnO NPs depends on their size [66]. The NP translocation coefficient values decreased with increasing ZnO NP size, amounting to 0.68 for 10–30 nm NPs, 0.55 for 80–200 nm NPs, and 0.27 for 300 nm ZnO NPs at the highest exposure concentration. When exposed to small NPs, they were detected in leaf margin tissue, while when exposed to large NPs, they were observed only in leaf veins. The authors report that large NPs diffused more slowly from the roots to the leaves of plants [66].

**Figure 2:**
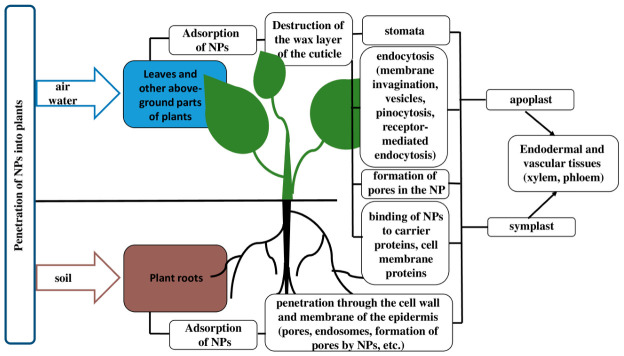
Penetration of nanoparticles (NPs) into plants.

Further interaction of NPs with the plant organism occurs upon contact of NPs with the plant cell wall, penetration of NPs into plant tissues and cells. The mechanisms of NP penetration into plant tissues and cells are actively studied. It has been found that NPs penetrate plant cells through endocytosis, accumulating in plant tissues [67,68]. NPs penetrate the cell by forming a vesicle, which can move to different parts of the cell [61]. It has been shown that after treating plants with ZnO and CuO NPs, endosomes (∼900 nm and ∼500 nm) containing NPs were found on the roots [60]. Some NPs are capable of forming pores for penetration into cells [61]. NPs can bind to carrier proteins and cell membrane proteins, which act as carriers for the intracellular penetration of NPs into plant cells [61]. It has been shown that the method of penetration of CuO NPs into plants depends on their genetic status. For example, CuO NPs aggregated on the epidermis (especially in the root area) of non-transgenic cotton plants, while in transgenic plants, CuO NPs penetrated cells via endocytosis [69] (Figure 2).

Thus, NPs penetrate the plant organism through soil, air, and water. The method of NP penetration depends on plant characteristics, the properties of the NPs themselves, and the method of their application: through stomata, by endocytosis, or by binding to carrier proteins. NPs move through plant tissues via two pathways: apoplastic and symplastic. NPs can accumulate in the epidermis, cortex, endodermis, cambium, and xylem of plants. After NPs penetrate plant tissue, they exert a biological effect, which may vary depending on the NP type, size, shape, and concentration.

## 4. Dose-Dependent Effect of MONPs

When describing the potential use of NPs in agrochemistry, several authors warn that excess or inappropriate NMs formulations can cause phytotoxicity and oxidative stress in plants [10,70]. Therefore, determining the safe eustress dose range is crucial [2]. Experimental selection of safe ranges and NMs application methods is recommended to minimize risks and maximize beneficial effects. This chapter presents studies of the effects of various MONP concentrations on plants.

### 4.1. CuO NPs

The biological effect of CuO NPs is largely related to their concentration. A dose-dependent effect of CuO NPs on woody plants has been reported. For example, application of 100 mg/kg CuO NPs to red loamy soil in forested areas significantly accelerated the growth of willow (*Salix fragilis* L.), while increasing the concentration to 500 mg/kg significantly slowed its growth [71]. Treatment of sweet cherry (*Prunus avium* L.) seeds after stratification with CuO NPs (38 nm) at concentrations of 400, 600, 800 and 1000 mg/L resulted in slow growth of sweet cherry seedlings, while treatment at a concentration of 200 mg/L improved the biometric characteristics of plants [72].

A dose-dependent effect of CuO NPs on plant growth and seed germination efficiency of mustard (*Brassica nigra* L. W.D.J. Koch) was demonstrated at various concentrations (250, 300, and 1000 mg/L). Concentrations of 250 and 300 mg/L CuO NPs increased seed germination efficiency and seedling length of mustard without accumulating in plant tissues. The highest Cu concentration in xylem and phloem was recorded at a concentration of 1000 mg/L and amounted to 2.2 and 5.7 µg/mL, respectively, compared to the control (1.0 and 4.9 µg/mL). The effect of CuO NPs on plasma membrane integrity and the rate of potassium ion (K^+^) leakage from mustard roots and shoots was significantly higher in roots treated with NPs at a concentration of 100 mg/mL than in the control [73]. It was also shown that when mustard foliage was treated with CuO NPs at different concentrations (0, 2, 4, 8, and 16 mg/L), CuO NPs at a concentration of 8 mg/L increased chlorophyll (Chl) content, photosynthesis rate, proline content, and activity of antioxidant enzymes (AOEs) [74].

Spherical CuO NPs (32–76 nm) at concentrations of 100 and 200 mg per kg of soil increased the nutritional value of cucumber (*Cucumis sativus* L.) fruits and elevated the potassium (K), Zn, phosphorus (P), and Mn contents. The biomass of aboveground parts also increased significantly (by 19.0%) at a concentration of 100 mg/kg. However, at a concentration of 1000 mg/kg after 15 days, CuO NPs were toxic to cucumber and maize (*Zea mays* L.) plants. Cu accumulated in plant tissues and reduced their growth [75].

A dose-dependent effect of CuO NPs on weedy and cultivated rice (*Oryza sativa* L.) plants was demonstrated in [76]. At a soil concentration of ≥300 mg/kg, rice did not produce grains. At a soil concentration of 75 mg/kg CuO NPs reduced grain yield by 38.3% compared to the control, and the Cu content in grains was higher than in the control. In weed-infested rice grains, the contents of K, magnesium (Mg), Zn, and calcium (Ca) decreased with soil concentrations of 75 and 150 mg/kg. This effect was not observed in cultivated rice, and the iron (Fe) content actually increased. In rice ears, CuO NPs increased the contents of Mg, Ca, Fe, and Zn. CuO NPs at concentrations of 75 and 150 mg/kg increased the transcription of an auxin-related gene in both weedy and cultivated rice grains [76].

Another study on rice (*O. sativa*) grown in a hydroponic system with a NPs solution showed that CuO NPs (<50 nm) at concentrations up to 50 mg/L stimulated plant growth and development by increasing activity of AOEs. CuO NPs at concentrations greater than 100 mg/L led to the development of oxidative stress [77].

The mechanism of the dose-dependent effect of spherical CuO NPs (35.6 nm) was discovered during a study of their treatment of lettuce (*Lactuca sativa* L.) roots [78]. A CuO NP concentration of 5 mg/L enhanced the functioning of signaling pathways and promoted pectin degradation under the influence of hydrogen peroxide, which led to loosening of the root cell wall and, consequently, root elongation. A CuO NP concentration of 450 mg/L affected the activity of xyloglucan endotransglycosylase/hydrolase (XTH) enzymes and caused a disordered distribution of pectin homogalacturonan, thereby increasing adhesion and strengthening the cell wall, which hindered root elongation. An apoplastic barrier was also formed, causing root lignification. These processes blocked the uptake of CuO NPs by roots. Endodermal cells had thickened cell walls, which significantly increased their ability to retain CuO NPs [78].

### 4.2. Mn_x_O_x_ NPs

MnO_x_ NPs, which have a needle-shaped shape with an average length of 80 nm and a width of 7 nm, after 5 days of exposure to seeds affected the growth rate of lettuce seedling roots depending on the NP concentration [79]. At a concentration of 10 mg/L, an increase in seedling length by 41.6% was observed, at 5 mg/L—by 53.9%, at 0.5 mg/L—by 14.7%, and at 0.025 mg/L—no significant increase was observed [79].

A dose-dependent effect is not always evident in biometric parameters; sometimes, the influence is only evident in biochemical characteristics. For example, treatment of watermelon (*Citrullus lanatus* (Thunb.) Matsum & Nakai) seeds with oval-shaped Mn_2_O_3_ NPs (22–39 nm) biosynthesized from bulb extract did not significantly affect seed germination or root elongation at MnO NP concentrations of 10–80 mg/L [80]. At a MnO NP concentration of 20 mg/L, Chl *a* and *b* levels significantly increased compared to control seedlings without priming and with hydropriming. MnO NPs at concentrations below and above 20 mg/L did not affect Chl *a* and *b* levels [80]. Differences in Chl *a* and *b* profiles between diploid and triploid varieties may be due to genetic makeup and seed morphology [80]. At the highest MnO NP concentration (80 mg/L), antioxidant activity levels were elevated. MnO NPs also significantly modulated the hormonal status of watermelon plants [80]. For example, at concentrations from 20 to 80 mg/L, a significant decrease in 12-oxophytodienic acid levels and an increase in jasmonic acid levels were observed in diploid watermelon seedlings. At MnO NP concentrations of 40 and 80 mg/L, triploid seedlings showed significant increases in abscisic acid and gibberellic acid levels, while salicylic acid and zeatin levels were significantly decreased. These results demonstrate that higher dose of MnO NPs can induce oxidative stress in watermelon plants [80].

Belladonna (*Atropa belladonna* L.) seedlings were grown in individual culture vessels containing 120 mL of Murashige–Skoog (MS) medium supplemented with Mn_2_O_3_ NPs (30 nm) [81]. A concentration of 25 mg/L Mn_2_O_3_ NPs stimulated the growth of belladonna and enhanced the biosynthesis of secondary metabolites by activating specific AOEs [81]. Increasing the concentration to 50–200 mg/L Mn_2_O_3_ NPs resulted in oxidative stress and toxicity (in terms of cellular damage markers) [81]. When applying a solution of MnO NPs to the leaves of tomato (*Solanum lycopersicum* L.), an increase in antioxidant protection was shown at a MnO NP concentration of 10 mg/L. With an increase in the concentration to 50 mg/L MnO NPs, oxidative stress was observed, which led to a decrease in the morphological and biochemical parameters of tomato [82].

A concentration of 50 mg/L MnO_2_ and Mn_3_O_4_ NPs (32 nm and 77 nm, respectively) applied to the soil promoted root elongation and increased seed germination in radish (*Raphanus sativus* L.). Even a lower concentration of 10 mg/L MnO_2_ and Mn_3_O_4_ NPs increased the height of radish plants compared to the control. A concentration of 100 mg/L MnO_2_ and Mn_3_O_4_ NPs led to an increase in the amount of malondialdehyde (MDA) in radish shoots. Furthermore, it was demonstrated that the application of Mn oxide-based NPs inhibits the uptake of essential metallic mineral elements by plants, such as Cu, Fe, Mg, Zn, sodium (Na), and K [83].

### 4.3. ZnO NPs

The biological effect of ZnO NPs on plants was dose-dependent. Seed treatment with spindle-shaped ZnO NPs (45 nm) positively affected the root length of rapeseed (*Brassica napus* L.) seedlings at concentrations up to 10 mg/L [84]. The maximum increase in root length was observed with ZnO NPs at a dose of 5 mg/L. At concentrations >10 mg/L, ZnO NPs inhibited rapeseed root growth. At a concentration of 50 mg/L, ZnO NPs reduced root growth and also demonstrated a negative effect on overall germination, radicle length, and plumule length (the first bud of the embryonic shoot in the seed). ZnO NP concentrations in the range of 0.1–10 mg/L were optimal for achieving the maximum viability index of rapeseed seedlings [84]. Similar results were obtained with the effect of smaller ZnO NPs (quasi-spherical, ∼8 nm) on rapeseed and mustard seeds (*B. juncea* L. Czern.) [85]. At a concentration of 25 mg/L, ZnO NPs had a positive effect on seedling growth, while at a concentration of 100 mg/L, they had a toxic effect, most likely due to penetration into root cells due to their small size (∼8 nm), which led to the release of Zn^2+^ ions and their subsequent increase in plant organs. Furthermore, ZnO NPs disrupted the homeostasis of superoxide radicals and hydrogen peroxide, and modulated the activities of metabolic enzymes (NADPH-oxidase, superoxide dismutase (SOD), ascorbate peroxidase (APX)) and non-enzymatic antioxidants (ascorbate and glutathione), causing similar changes in oxidative signaling in plants [85].

Seed treatment with polycrystalline, spherical, non-aggregating ZnO NPs (35 nm) synthesized using aloe vera (*Aloe barbadensis* Mill) leaf extract resulted in significant increase in shoot and root length of wheat (*Triticum aestivum* L.) at 62 mg/L compared to other concentration levels (15, 125, 250, and 500 mg/L) [86]. Other concentrations ZnO NPs (32 nm) were tested on wheat in another study [87]. When wheat seedlings were grown hydroponically using a ZnO NP concentration of 1 mg/L, an increase in Chl *b* content and root growth was observed, whereas a concentration of 2–4 mg/L resulted in a decrease in root growth [87].

On days 45 and 60 after treatment of tomato seedlings with 10 mg/L ZnO NPs for 30 min, improved growth and photosynthesis performance, increased activity of various AOEs (catalase (CAT), peroxidase (POD), and SOD), and higher proline and protein contents were observed compared to untreated plants [59]. At higher ZnO NP concentrations (100 mg/L and 200 mg/L), the values of all studied parameters decreased. Thus, root treatment with 10 mg/L ZnO NPs was optimal for most of the evaluated parameters.

The effect of foliar application of spherical ZnO NPs (29 nm) synthesized using *Citrus medica* L. peel extract demonstrated the highest values of seed germination, root length, shoot length, fresh shoot weight, total dry weight, number of branches, pods, and leaf area in *Abelmoschus esculentus* L. when treated with a low concentration of ZnO NPs (20 mg/L) [88]. The lowest studied parameters were recorded at ZnO NP concentrations ranging from 50 to 200 mg/L.

Irrigating tomato seedlings with suspensions containing 200 mg/L ZnO NPs increased AOE activity and transcription of the corresponding genes, suggesting that ZnO NPs could enhance the defense response by increasing activities of AOEs, but had no significant effect on plant growth or photosynthetic parameters [89]. Moreover, ZnO NPs exhibited toxicity when concentrations were increased to 400 and 800 mg/L. At a concentration of 400 mg/L, a 10% decrease in shoot and root dry weight was observed, while 800 mg/L treatment resulted in a 50% inhibition. Effects on Chl content and photosynthesis were also observed. In another study, the stimulating dynamics of tomato plant growth parameters after 30 min immersion of roots in a ZnO NPs solution was observed for much lower concentrations in the following ascending order: 8 > 16 > 4 > 2 > 0 mg/L [90].

Treatment of maize seeds with different concentrations (2, 4, 8, 16 mg/L) of spherical ZnO NPs (16–20 nm) biosynthesized using *Bacillus* spp. showed the highest increase in root and shoot length and protein content (77.3%) in the plants at a concentration of 8 mg/L. The general pattern for growth parameters and protein content in treated plants at different concentrations was as follows (from highest to lowest): 8 > 16 > 4 > 2 > 0 mg/L ZnO NPs [91].

The dose-dependent biological effect of ZnO NPs under abiotic stress conditions was studied in [92]. *Coffea arabica* L. seedlings were grown on acidified soil sprayed with ZnO NPs at concentrations of 10, 25, 50, and 100 mg/L. At concentrations of 10–25 mg/L, ZnO NPs had no effect on the rate of photosynthesis, and ionic balance was maintained. At a concentration of 100 mg/L, the amount of H_2_O_2_ in plant tissues increased and photosynthesis deteriorated [92]. The same authors studied the role of ZnO NPs in *C. arabica* under NaCl salinity conditions [70]. Treatment of leaves with ZnO NPs at a concentration of 50 mg/L resulted in an increase in proline content, an increase in CAT activity, an accumulation of Na^+^ ions, and a decrease in the amount of H_2_O_2_. ZnO NPs at a concentration of 100 mg/L and NaCl led to a decrease in carotenoid content [70].

In a hydroponic experiment on wild apple (*Malus robusta*), foliar application of 200 mg/L of a commercial ZnO NP (30 nm) preparation to plants demonstrated a positive effect on the biometric and biochemical characteristics of the plants. Higher concentrations (500 and 1000 mg/L) induced oxidative stress but did not inhibit apple growth. The authors attributed this effect to the strong antioxidant activity of ZnO NPs and nutrient binding [93].

The dose-dependent biological effects of MONPs described above are briefly summarized in Table 1.

## 5. Dependence of the Biological Effect of MONPs on Shape and Size

### 5.1. CuO NPs

When cucumber (*C. sativus*) was grown for 65 days in soil with the addition of CuO NPs (43 nm) and CuO microparticles (μCuO, 510 nm) to the soil at a concentration of 200 mg/kg, the Cu content in shoots, fruits and leaves significantly increased. Moreover, the Cu content in leaves treated with CuO NPs at a concentration of 50 mg/kg was 1.576 times higher than that treated with μCuO microparticles. The Cu content in shoots treated with CuO NPs at a concentration of 100 mg/kg and 200 mg/kg was 32.82% and 25.14% higher, respectively, than that treated with μCuO microparticles [94]. An earlier study also showed that CuO NP treatment of soil resulted in a greater increase in Cu content in *Brassica rapa* roots than μCuO microparticle treatment. Moreover, the Cu translocation ratio in all CuO treatments was significantly higher compared to the control, demonstrating the high transport capacity of both μCuO microparticles and CuO NPs. The translocation ratio of CuO NP treatment was 33.04% higher than that of μCuO treatment at the concentration of 200 mg/kg. Overall, CuO NPs could increase the Cu content in cucumber with the increasing concentration of CuO NPs in soil [95]. In a similar study, *Lemna minor* was more affected by CuO NP (40 nm) treatment, even at a low concentration (10 mg/L), than by bulk CuO particles (10 μm). Also, the highest increase in AOP activity was observed after NP treatment [96]. Using bio-optical computed tomography, it was possible to quickly and safely monitor the internal activity of seeds and seedlings during growth. Seed treatment at concentrations of 25 and 100 mg/L and foliar treatment at a concentration of 100 mg/L of lentil (*Lens culinaris* L.) with Cu NPs (25 nm) and CuO NPs (<50 nm) resulted in a significant decrease in biospeckle contrast, indicating increased physiological stress, while exposure to microparticles (<10–25 μm) had a minimal or even positive effect. Thus, treatment with CuO NPs resulted in a marked decrease in the activity of nucleotide-binding proteins, indicating an inhibitory effect on physiological processes in seeds. The inhibitory effect of CuO NPs on the growth of lentil seedlings increased with an increase in concentration to 100 mg/L [97].

When growing soybean (*Glycine max* cv. Kowsar) in soil with three sizes of CuO NPs (25, 50, 250 nm), the lowest seed yield was obtained at a CuO NP (25 nm) concentration of 500 mg/kg in soil. The highest seed yield (19.13 g/plant) was obtained in control plants without NP treatment, while it was almost no different from the yield obtained when treated with CuO NPs (50 nm) or Cu ONPs (250 nm) at a concentration of 50 mg/kg. The effect of CuO NPs (25 nm) on antioxidant enzymes was significantly stronger than that of the two larger CuO NPs tested (50 and 250 nm) [98]. Thus, the results demonstrated that the inhibition of CuO NPs (25 nm) was significantly higher than that of the two larger CuO NPs at all concentrations tested. In the study by Guo et al. [99] soybean seeds were germinated and grown in soil fertilized with different doses (1, 5, 10, 25, and 50 mg/kg) of CuO NPs of two particle sizes. At a concentration of 10 mg/kg, CuO NPs (50 nm) significantly increased the fresh biomass of soybean compared to CuO NPs (20 nm). Only CuO NPs of 50 nm size at a concentration of 10 mg/kg had a positive effect on the increase in the fresh weight of roots. The activity of enzymes associated with nitrogen assimilation and the content of nitrogenous compounds, including nitrates, proteins, and amino acids, in soybean tissues increased significantly with all treatment options with CuO NPs. The applied doses of CuO NPs of two sizes did not affect the Cu content in shoots and roots, but increased the Cu content in the soil in a dose-dependent manner [99].

In contrast to the studies presented above, it was shown that an increase in the concentration of CuO NPs with a particle size of 20–26 nm, introduced into the soil, leads to an increase in the length of shoots and roots of *Z. mays*. A significant elongation of plant shoots and roots was observed for all used concentrations of CuO NPs—300, 550 and 800 mg/L. Moreover, the greatest increase in shoots (25%) and roots (21%) was observed at a concentration of 800 mg/mL. The highest SOD activity in shoots was observed at a concentration of 550 mg/mL, with an increase in concentration to 800 mg/mL a sharp decrease in SOD levels was observed. Similar results were obtained for the activity of CAT and POD [100].

### 5.2. Mn_x_O_x_ NPs

Exposure to Mn NPs in the soil had subtle effect on plants, differing little from the effect of bulk or ionic manganese. Thus, 4 weeks after germination of winter wheat (*T. aestivum*) seeds, spherical Mn_2_O_3_ NPs with a particle size of 30 nm were applied to the soil at a concentration of 6 mg/kg and to the seedling (foliar treatment), along with bulk Mn_2_O_3_ oxide and manganese chloride (MnCl_2_ × 4H_2_O) for comparison [101]. At this exposure dose, no signs of overt toxicity to the crop were observed. All types of Mn significantly (by 9–18%) reduced the nitrogen content in wheat shoots. Despite this, Mn NPs led to an increase in the efficiency of manganese transfer to the grain (by 22%) compared to manganese salt (by 20%), bulk manganese (by 21%), and the control (by 16%). These results indicate that exposure to Mn_2_O_3_ NPs in soil may have subtle effects on plants that differ from those of bulk or ionic manganese [101].

Treatment of isolated mung bean (*Vigna radiata*) chloroplasts with small 20 nm Mn NPs at low concentrations of 0.05, 0.1, 0.5, and 1 mg/L showed higher photophosphorylation activity and oxygen evolution capacity compared to control samples [102]. Also, the activity of water splitting and CP43 protein (which is a part of the photosystem II complex) was significantly increased compared to control samples. The authors reported that Mn NPs did not cause oxidative stress or phytotoxicity [102]. Mung bean treatment with Mn NPs resulted in enhanced nitrogen metabolism, since the expression and activity of nitrogen reductase protein were increased under the influence of Mn NPs compared to the control sample [103].

### 5.3. ZnO NPs

The biological effect of ZnO NPs (spherical shape with a size of 30 and 50 nm, columnar shape with an average size of 45–90 nm, and in the form of hexagonal rods with a size of 100–150 nm) depends on their shape and size. For example, studies showed that spherical ZnO NPs with a diameter of up to 30 nm significantly slow down seed germination and plant growth compared to ZnO larger NPs [104]. However, even exposure of onion (*Allium cepa* L.), tobacco (*Nicotiana tabacum* L.), and common bean (*V. faba*) to ZnO NPs (aggregates of cuboid, hexagonal-octagonal and rod-shaped particles with a size of 75–85 nm) with a diameter of ∼85 nm resulted in loss of membrane integrity, an increase in the number of chromosomal aberrations, the formation of micronuclei, DNA strand breaks, cell cycle arrest, increased production of intracellular reactive oxygen species (ROS), intensification of lipid peroxidation (LPO), and an effect on the activity of some AOEs [105].

Pérez Velasco et al. [106] studied two application methods (foliar and watering) of spherical (22.5 nm) and hexagonal (85 nm) ZnO NPs (in spherical <30 nm and hexagonal 60–120 nm shapes) with and without maltodextrin (MD) surface modification at a concentration of 1500 mg/L on tomato growth. Hexagonal ZnO NPs increased stem diameter. Surface modification and the application method did not affect this parameter. Plant height was higher regardless of the application method when using MD-modified NPs. Spherical NPs without surface modification also increased stem diameter after foliar application, while MD surface modification did not have this effect. Hexagonal NPs modified with MD increased plant height, while treatment of control plants with MD alone without NPs also produced this effect. It is likely that the polysaccharide MD, obtained by starch hydrolysis and used to modify the NPs, acts as a plant growth stimulant. This effect was enhanced when used in combination with ZnO NPs. When using NPs alone, regardless of the form and method of application, a decrease in stem dry weight was observed. Watering tomato with only MD added resulted in an increase in stem dry weight [106]. Thus, despite the high concentration of NPs used, modifying the particle surface with polysaccharide has a beneficial effect on plants, reducing their toxicity.

Most studies provide compelling evidence that the toxicological effects of nanoparticles (NPs) differ significantly depending on their size, with smaller NPs generally exhibiting greater phytotoxicity than larger ones; however, this relationship must be considered in conjunction with NP concentration. Overall, the biological effects of MONPs (CuO, Mn_x_O_x_, and ZnO NPs) are governed by a combination of concentration, size, and shape, and can vary markedly among plant species, likely due to interactions with plant macromolecules [107]. The variability in plant responses to different NP characteristics highlights the need for case-specific evaluation of nanomaterials, as even the same species may respond differently to specific NPs, complicating the development of standardized application protocols. It should also be noted that data on the dose-dependent effects of Mn_x_O_x_ NPs remain more limited compared to CuO and ZnO NPs. Depending on the applied concentration, MONPs can exert both beneficial and adverse effects on plant growth and development.

## 6. Phytotoxicity of MONPs

The negative impact of NPs on plant growth is primarily caused by their excess [108,109]. The mechanism of NP toxicity to plants is currently unclear, but it is believed to involve, firstly, their ability to overcome the permeable membrane barrier, seed coat, and roots [110]. The cell wall is considered the main barrier to NPs. Thus, when seeds are treated, NPs penetrate the seeds both passively with water and through damaged areas of the seed coat [111]. The ability of NPs to concentrate on the surface of plant roots, altering the chemical composition of the root surface and, as a result, leading to structural and functional disturbances, reducing hydraulic conductivity and inhibiting transpiration, as well as affecting the absorption of water and nutrients by plant roots [112,113]. Venzhik et al. [111] suggested that positively charged NPs are actively absorbed only by plant roots, while negatively charged ones are able to effectively move from the roots to the stems and leaves.

Within plants, NPs can interact with proteins, lipids, and carbohydrates, forming an “organic corona” around themselves that facilitates the overcoming of biological barriers [111]. Some NPs can be transported and accumulate in plants, leading to phytotoxicity [113]. The release of metal ions from metal NPs and MONPs suspensions is inevitable. Released metal ions are believed to be a significant factor in their phytotoxicity [60].

The mechanisms of toxic effects of NPs on plants can be briefly summarized as follows: (1) NPs interact with plants to increase ROS production, leading to oxidative damage [108,114]; (2) NP-induced transcriptional disruption in plants [115]; (3) NPs interact with DNA or organelles (e.g., mitochondria), leading to genotoxicity [116]. Atha et al. [117] reported DNA damage resulted from NP interaction with plants. Metallic NPs activate genes involved in defense mechanisms, leading to significant oxidative stress in target plants [117]. NPs can affect plant anatomy and ultrastructure after physically interacting with transport pathways in plant cells. They can block the apoplastic or symplastic pathway, thereby affecting plant water exchange or nutrient uptake [118]. In response to NPs exposure, ROS generation, cell membrane disruption, increased LPO in membranes, and thereby suppression of plant metabolism and growth occur [119,120]. Furthermore, transgenerational effects of NPs have been reported [121].

The impact of NPs on plants is determined by their absorption, accumulation in plant tissues and organs, and subsequent transport to various parts of the plant [53,122]. However, these three processes depend on the physicochemical properties of the NPs (chemical composition, structure, size, surface area, etc.), as well as the plant genotype and anatomy.

This review focuses primarily on the effects of MONPs (CuO, Mn_x_O_x_, and ZnO NPs) on plants. Therefore, the negative effects of these NPs on plants will be described below.

### 6.1. CuO NPs

The phytotoxic effects of CuO NPs have been extensively studied in the literature on cereals (Poaceae). For example, in maize, a CuO NP concentration of 500 mg/kg reduced the dry biomass of shoots and roots [123]. Adding CuO NPs to a hydroponic rice cultivation medium suppressed seedling growth, increased MDA content, affected AOE activity, and reduced the amount of photosynthetic pigments [124].

The negative effects of CuO NPs on wheat have been repeatedly demonstrated [125,126,127,128]. The toxicity of CuO NPs increased with increasing plant cultivation temperature, which inhibited root growth [128]. The addition of CuO NPs to the hydroponic wheat cultivation medium inhibited seedling growth, reduced plant biomass and total Chl content, increased the amount of MDA in shoot and root tissues, and increased AOE activity [127]. In another study, the authors attributed the decrease in shoot growth under the influence of CuO NPs and a decrease in grain yield to starch degradation [125].

The negative effects of CuO have been demonstrated on barley plants [129,130,131]. CuO NPs at high doses (2000 and 10,000 mg/kg) when applied to the soil inhibited the growth of spring barley (*Hordeum sativum* L.). CuO NPs accumulated in plant tissues and formed complex metal compounds with organic matter [129]. In another study, adding 1320 mg/kg CuO NPs (30–50 nm) to sandy fluvisols and stagnant fluvisols of humus-rich heavy loamy soils resulted in shortening of shoots and roots in common barley (*Hordeum vulgare* L.) [131]. Increases in the Cu content in shoots and roots, MDA in roots by 52%, and in shoots by 46% were also observed after the addition of CuO NPs compared to control plants, as well as a 6-fold increase in CAT activity in shoots and SOD activity in shoot and root tissues of barley [131]. CuO NPs induced changes at the cellular and tissue levels in the roots of *Hordeum sativum distichum* L. [130].

Data on the phytotoxicity of CuO NPs on representatives of other families, including Malvaceae, Brassicaceae, Cucurbitaceae, Apiaceae, as well as woody and aquatic plants, have also been obtained. For example, soil contamination with CuO NPs at a concentration of 1000 mg/kg proved toxic to cucumber plants after 15 days. CuO NPs accumulated in plant tissues and reduced their growth [75]. Exposure to CuO NPs at a dose of 50 mg/kg decreased the rate of photosynthesis and transpiration compared to the control. Increasing the dose to 100 and 200 mg/kg significantly increased the transpiration rate compared to the control, which may be the reason for the acceleration of the adsorption and movement of metal elements [75]. Another study showed that CuO NPs did not affect the MDA content in cucumber tissues [94].

CuO NPs caused a decrease in Chl and carotenoid content and an increase in proline and anthocyanin content in turnip (*B. rapa* L.) plant tissues, indicating plant stress [125]. In common cotton (*Gossypium hirsutum* L.) plants, CuO NPs delayed growth and development due to a decrease in hormones and nutrients [69]. Soil treatment with CuO NPs led to a decrease in carotenoid and total Chl content in parsley (*Petroselinum crispum* (Mill.) Fuss) plant tissues and the accumulation of CuO NPs in the roots [132]. Seed germination, root development, and photosynthesis of the tropical tree *Sesbania virgata* (Cav.) Poir. were inhibited by CuO NPs [133]. A negative effect of CuO NPs on photosynthesis was also observed in duckweed (*Lemna valdiviana* Phil.) [134].

Thus, the negative effects of CuO NPs manifest themselves both in their impact on the biometric characteristics and at the physiological and biochemical levels of various plants. This manifests itself in their effects on the components of the photosynthesis process and the functioning of the antioxidant system.

### 6.2. Mn_x_O_x_ NPs

Mn_2_O_3_ NPs inhibited the germination and subsequent growth of *Artemisia annua* L. plants both when applied as presowing seed treatment and in combination with foliar feeding [135]. The phytotoxicity of Mn_3_O_4_ NPs on ground moss (*Physcomitrella patens* (Hedw.) Bruch & Schimp.) plants was demonstrated [116]. Exposure to 20 μg/mL Mn_3_O_4_ NPs induced a surge in ROS, causing DNA hypomethylation in *P. patens* gametophores [116].

### 6.3. ZnO NPs

The toxic effect of Zn-containing NPs was manifested at the biochemical level and morphometric parameters of plants. In *Brassica* species, a disruption of the antioxidant system was demonstrated under the influence of ZnO NPs [85]. Changes in the homeostasis of reactive nitrogen species (nitric oxide, peroxynitrite, and S-nitrosoglutathione) were noted. ZnO NPs also caused changes in protein carbonylation and nitration. These effects indicate that ZnO NPs cause changes in the nitrooxidative signaling pathway, which may contribute to the toxicity of ZnO NPs through oxidative stress [85]. Other studies on the long-term effects of ZnO NPs (<50 nm) on rapeseed plants also confirm these results [112]. Treatment of rice with ZnO NPs (25, 50, and 100 mg/L) under hydroponic conditions inhibited seedling growth and reduced their biomass compared with treatment with Zn^2+^ ions (13.82 mg/L) and the control (no treatment) [60]. In rice leaves treated with ZnO NPs at concentrations of 25, 50 and 100 mg/L, toxic effects were also found, associated with a decrease in the content of Chl *a* (by 48.5%, 67.5% and 76.7%) and Chl *b* (by 53.8%, 70.5% and 75.7%), respectively to concentration, and oxidative damage expressed in the change in the expression level of genes encoding AOEs (CAT, ascorbate oxidase, and SOD) [60]. The use of spherical ZnO NPs (27.5 nm) at concentrations of 150 and 200 mg/L on wheat and maize seeds led to a decrease in the biometric characteristics of plants, as well as a decrease in the activity of α-amylase and dehydrogenase in plants compared to the control. Zn accumulated more in the roots [136]. Biochemically, it was shown that high Zn accumulation overloaded plants, disrupted Zn homeostasis, and led to an increase in MDA levels, as well as a decrease in AOE activity and plant growth [136]. The phytotoxicity of ZnO NPs (300 nm) at a concentration of 1000 mg Zn/kg manifested itself as a decrease in leaf and root weight of *Brassica chinensis* L., a decrease in Chl *a* and *b* content, and an increase in oxidative stress [66].

Thus, the negative impact of ZnO NPs is associated with the development of oxidative stress, characterized by increased MDA levels and changes in the activity of AOEs, amylase, and dehydrogenase in plant tissues. These effects lead to a decrease in plant biometric parameters and photosynthetic pigments.

## 7. MONPs as Potential Eustressors in Plants

### 7.1. CuO NPs

Cu is an essential nutrient required for plant growth, photosynthesis, enzyme function, and RNA (ribonucleic acid) synthesis. In the form of copper sulfate (CuSO_4_) solution, Cu is widely used in agriculture as a fungicide, algicide, herbicide, and even a disinfectant [73,137,138]. However, the use of Cu as a micronutrient in the form of NPs is very promising, as demonstrated by the data presented below.

#### 7.1.1. Stimulating Effect of CuO NPs on Morphometric and Biochemical Parameters of Stress-Resistant Plants

Encouraging results have been obtained on cereals. It was shown that soil treatment with CuO NPs (28 nm) at 50 mg/kg significantly increased Cu concentration in wheat shoots and improved photosynthesis [139]. Also, a stimulating effect of CuO NPs at a concentration of 0.01 g/L on germination and morphometric parameters of seedlings was shown on wheat [140]. Treatment of rice seeds with CuO NPs stimulated the growth and development of seedlings under hydroponic conditions [141]. The addition of 10 mg/L CuO NPs to Murashige–Skoog medium improved organogenesis and the overall growth intensity of *Stevia rebaudiana* (Bertoni) Bertoni plants [142]. Adding CuO NPs to a hydroponic environment increased the biomass, length, and number of roots in Chinese cabbage (*Brassica chinensis* L.), and increased the amount of beneficial elements in plant tissues [143]. Seed treatment with CuO NPs increased germination and vigor in cowpea (*Vigna radiate* L.) [144] and lettuce [145].

Tamil Elakkiya et al. [73] investigated the effect of CuO NPs on plant growth and seed germination efficiency of mustard (*B. nigra*). Concentrations of 250 and 300 mg/L CuO NPs increased seed germination efficiency and seedling length of mustard. Cu accumulation in xylem and phloem after CuO NPs treatment was comparable to the control [73]. Spraying with CuO NPs increased shoot biomass of a herbaceous plant *Dracocephalum moldavica* L. compared to the control in [146]. Four treatments with Cu NPs (50, 125, 250, and 500 mg/L; 50 nm) were applied twice during tomato development to evaluate their effects on tomato fruit quality and antioxidant compounds in [147]. Cu NP treatments increased fruit firmness, vitamin C, lycopene content, and ABTS antioxidant capacity compared with the control. Enzymatic activity of APX and glutathione peroxidase (GPX) decreased, whereas SOD and CAT activities significantly increased. Overall, Cu NPs promoted the accumulation of bioactive compounds in tomato fruits [147]. Furthermore, CuO NPs stimulated the growth and development of various trees [148,149,150].

#### 7.1.2. The Effect of CuO NPs on Plant Stress Resistance

Due to their sedentary lifestyle, plants can be simultaneously exposed to a large number of different stress factors. These factors negatively impact the physiological and biochemical state of plants, their morphometric characteristics, and yield. Some authors believe that NPs in eustress concentrations can mitigate the effects of stress factors [2,16,36]. The mechanism of these effects is explained by a beneficial effect on plant growth and development, productivity, activation of antioxidant system components, and the accumulation of secondary metabolites that promote plant resistance [2]. This section will present information on the effect of CuO NPs on the physiological, biochemical, and morphometric characteristics of plants subjected to stress.

There is evidence of a positive effect of CuO NPs on plant resistance to biotic stress. For example, tomato infected with the soil-borne bacterial plant pathogen *Ralstonia solanacearum* were grown in soil treated with 500 mg/kg spherical CuO NPs (20–39 nm) in [151]. Treatment with CuO NPs significantly improved the growth of tomato affected by brown spot, mitigating the effects of the disease compared to the control. The use of CuO NPs led to an increase in tomato plant length by 24.3%, fresh weight by 32.8%, and dry weight by 41.0% compared to the control. The treatment reduced the MDA content by 16.9% and increased the POD and SOD activities in tomato plants by 21.5% and 59.6%, respectively [151]. In a greenhouse experiment, irrigating tobacco plants with CuO NPs enhanced their resistance to black rot by increasing the activity of AOEs (polyphenol oxidase, chalcone isomerase, phenylalanine ammonia lyase, and POD) in the root tissues of infected plants [152]. CuO NPs at a concentration of 40 ppm reduced the infestation of chickpea plants by the phytopathogens *Rhizoctonia solani* by 61% and *Fusarium oxysporum* f. sp. *ciceris* by 65%, increased plant yield, and stimulated plant growth. Biochemically, CuO NPs increased the activity of POD and polyphenol oxidase in chickpea plants and increased the phenolic content in plants [153]. Spraying potato plants with CuO NPs at a concentration of 100 mg/L in a greenhouse experiment demonstrated a 69% reduction in *Phytopytora infestans* infestation, while at a concentration of 50 mg/L, it was 46% lower than in the control [154]. Watermelon plants were sprayed with CuO NPs at a concentration of 500–1000 μg/mL in a greenhouse and planted in a soil mixture contaminated with *Fusarium oxysporum* f. sp. *niveum* in [155]. An increase in plant biomass and yield was observed, accompanied by the induction of the polyphenol oxidase and *PR1* genes [155]. ZnO and CuO NPs synthesized using hemp (*Cannabis sativa* L.) leaves (ZnONP-HL and CuONP-HL) were applied to soybeans (*G. max*) by foliar application as bionanofungicides against *Fusarium virguliforme* [156]. ZnONP-HL and CuONP-HL at a concentration of 200 μg/mL significantly (*p* < 0.05) increased (by approximately 50%) soybean growth compared to diseased control plants. NPs improved nutrient content (e.g., K, Ca, and P) and enhanced photosynthetic performance of plants by 100–200%. A 300% increase in the expression of *GmPR* genes, which are associated with soybean pathogenesis and encode antifungal and defense proteins, was observed; the biosynthesized NPs were shown to enhance resistance to fungal phytopathogens. The results of this study provide new evidence for the systemic suppression of fungal diseases by nanobiopesticides through stimulation of plant defense mechanisms [156].

**Figure 3:**
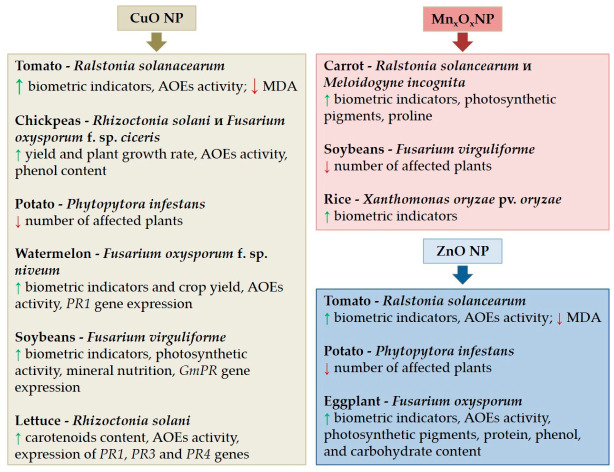
Effects of ZnO, CuO, and Mn_x_O_x_ NPs on phytopathosystems [151,152,153,154,155,156,157,158,159,160,161].; ↑—increased, ↓—decreased.

A study was conducted to evaluate the effectiveness of silicon dioxide (SiO_2_), CuO, and gamma Fe oxide (γFe_2_O_3_) NPs in inducing systemic resistance (SR) in lettuce against *R. solani* by examining the expression of pathogenesis-related genes [157]. Infected plants exhibited higher levels of LPO (MDA and H_2_O_2_). However, treatment with SiO_2_ and γFe_2_O_2_ effectively mitigated oxidative stress. All NPs increased carotenoid content and AOE activity (SOD, CAT, and APX), with γFe_2_O_2_ being the most effective. Importantly, NPs induced the expression of pathogenesis-related genes *PR1*, *PR3*, and *PR4*, as well as the ethylene-responsive transcription factor 1A (*ERT1*) gene. The upregulation of these genes correlated with a reduction in disease symptoms and improved physiological status, indicating that the increased gene expression contributed to the observed systemic resistance [157]. *PR1* gene expression was significantly increased by CuO NP exposure, *PR4* by γFe_2_O_2_ and SiO_2_ NPs, and *PR3* increased with all NPs. This study showed that NPs induced the expression of *PR1*, *PR3*, and *PR4* genes, suggesting that CuO, γFe_2_O_3_, and SiO_2_ NPs play a role in enhancing the plant immune response against *R. solani* [157]. The effects of ZnO, CuO, and MnO NPs on phytopathosystems are shown in Figure 3.

There are few studies on the effect of CuO NPs on plant resistance to abiotic stress factors. Thus, CuO NPs reduced cadmium (Cd) toxicity in bean plants *Phaseolus vulgaris* L. [162]. Biochemically, this was reflected in a decrease in ROS content, an increase in AOE activity, and a reduction in the activity of ROS-producing enzymes [162]. It was shown in vitro that treatment with CuO NPs can significantly suppress the negative impact of salt stress (50 mM NaCl) on alfalfa callus samples (*Medicago sativa* L.), leading to a decrease in the amount of ROS and AOF activity, and an increase in the protein content in plant tissues [163]. Thus, these published studies demonstrate that CuO NPs can be considered eustress agents for plants, as they can influence components of the antioxidant system and physiological processes in plants, activating plant growth and development even under biotic and abiotic stress.

### 7.2. Mn_x_O_x_ NPs

Mn is a minor micronutrient, but is nevertheless the second most important element for active plant growth and development [79,164,165]. It is essential for normal photosynthesis in plants. Mn deficiency is observed worldwide, especially in soils with high pH (>6.0) or in calcareous, sandy, peaty, or silty soils. The main problems associated with Mn deficiency are plant nutritional disorders. Therefore, the application of manganese-containing fertilizers is very important for increasing crop yields. To prevent these disorders, the use of Mn-based NPs as an alternative to MnSO_4_ for crop treatment has recently increased [51]. The diversity of Mn-containing NPs is represented by the following compounds: Mn, MnO, MnO_2_, Mn_2_O_3_, Mn_2_O_4_, Mn_3_O_4_, Mn_5_O_8_ and other Mn-based NPs synthesized with the participation of other metals [166,167].

#### 7.2.1. Stimulating Effect of Mn_x_O_x_ on Morphometric and Biochemical Parameters of Plants Not Exposed to Stress

It has been experimentally shown that the stimulating effects of MnO NPs (75 nm) on tomato growth were significantly higher than those of bulk MnO particles [82]. Foliar treatment of tomato with MnO NPs at a concentration of 10 mg/L increased root and shoot weight, root and shoot length compared to the control. An increase in several biochemical parameters was noted: Chl *a* and *b* content, total phenolic content in roots, and flavonoid content in both roots and shoots. A significant reduction in oxidative stress was observed: MDA levels in shoots and proline in roots decreased, indicating improved stress tolerance. Polyphenol oxidase activity increased in roots, and guaiacol peroxidase activity increased in shoots. These parameters reflect the activity of enzymes involved in the metabolism of phenolic compounds and protection against oxidative stress [82]. In a pot experiment on wheat, MnO NPs synthesized from a *Bacillus flexus* strain were shown to reduce oxidative stress and improve plant growth [168].

Foliar application of MnO_2_ NPs (diamond-shaped, 70 nm) to common bean *P. vulgaris* at a dose of 40 mg/L increased morphometric traits (plant and root length, leaf number, fresh and dry weight, and flower number) and yield over two seasons. A concentration of 40 mg/L increased the content of Chl *a*, *b*, and total Chl. A concentration of 20 mg/L NP resulted in the highest carotenoid content [169].

Sharma et al. [170] showed that the application of Mn-based NPs, including Mn, MnO, Mn_2_O_3_, MnO_2_, Mn_3_O_4_, MnFe_2_O_4_, Mn_0.5_Zn_0.5_Fe_2_O_4_, biochar-modified MnO_2_, and composite NMs such as chitosan/silver/Mn_0.5_Mg_0.5_Fe_2_O_4_ (Cs/Ag/MnMgFe_2_O_4_), under field conditions increased growth parameters by 45% and yield by 49% compared to untreated plants. NPs also modulated signaling, regulated the expression of stress-related genes, and activated defense mechanisms, thereby maintaining overall plant health and productivity [170]. It was shown that MnO NPs synthesized using *Syzygium cumini* L. leaf extract stimulated callus formation in *Moringa oleifera* Lam., prevented its infection and provided an optimal environment for rapid growth and development [171].

#### 7.2.2. The Effect of Mn_x_O_x_ on Plant Resistance to Stress

MnO_2_ NPs can increase plant resistance to biotic stress. For example, treatment of carrot leaves with MnO_2_ NPs (100 mg/L) in combination with *Pseudomonas putida* led to a significant increase in plant growth and an increase in the content of Chl, carotenoids, and proline in plant tissues affected by wilt caused by the bacteria *R. solancearum* and *Meloidogyne incognita* [158]. A single treatment of soybean plants by spraying them with Mn_2_O_3_ NPs (30 nM) at a high dose of 1000 mg/L reduced root rot damage caused by the phytopathogen *F. virguliforme* [159]. Moreover, NPs themselves did not suppress the growth and development of the pathogen, but exerted a stimulating effect by modulating plant nutrition [159]. Rice seedlings were treated with MgO and MnO_2_ NPs synthesized using a *Xanthomonas oryzae* pv. *oryzae* bacteriophage lysate [160]. MnO_2_ NPs stimulated plant growth during infection with *X. oryzae* pv. *oryzae* to a greater extent than MgO NPs [160].

The stimulating role of Mn oxide NPs on plants under abiotic stress has also been demonstrated. MnO NPs (18 nm) biosynthesized from *Conocarpus erectus* L. leaf extract improved physical growth parameters, Chl *a*, *b*, total Chl, and carotenoid content, and also increased SOD and POD activities in maize under drought conditions when foliar applied at a concentration of 75 mg/L [172]. Another study on maize in a field experiment also demonstrated the positive effect of foliar application of MnO NPs (20 ppm) in combination with ZnO NPs (100 ppm) under drought conditions [173]. NPs led to an increase in the rate and duration of seed filling, and also caused an increase in seed yield. In addition, treated plants showed increased green leaf area, leaf tissue Chl index, and proline content compared to water sprayed control [173].

Treatment of pepper (*Capsicum annuum* L.) seeds with Mn_2_O_3_ NPs (0.1, 0.5, and 1.0 mg/L) under salt stress significantly improved root growth in seedlings [174]. Scanning electron microscopy and energy-dispersive spectroscopy showed that Mn_2_O_3_ NPs penetrate the seed coat and form an NP-coat complex [174]. Foliar feeding of deciduous tree *Cyclocarya paliurus* (Batalin) Iljinsk with MnO_2_ NPs at a concentration of 50 mg/L under salt stress significantly increased the photosynthesis rate and seedling height, and reduced the salt damage index. In addition, NPs increased the expression of 50 photosynthesis-related genes [175].

There are many studies devoted to the study of the improvement of tolerance to heavy metals under the influence of Mn oxide NPs. Anar et al. [176] showed that the treatment of tomato seeds with tetragonal MnO NPs (22 nm) synthesized in *Bacillus subtilis* filtrate mitigated plant stress caused by lead and improved the growth performance of tomato seedlings. The concentration of 2.5 g/L MnO NPs showed the best results: a decrease in MDA, an increase in AOE activity, an increase in the content of osmolytes and concentration of photosynthetic pigments, and a decrease in lead accumulation in plant tissues [176]. In a similar study, positive effects of tetragonal MnO NPs (22 nm) synthesized using citrus peel *Citrus paradisi* Macfad. were also observed when treating wheat seeds with them [177]. Treated and untreated wheat seeds were grown in soil supplemented with lead (Pb) at concentrations exceeding the maximum permissible concentration. Treatment with MnO NPs significantly improved the morphological growth characteristics of wheat seedlings, as well as physiological and biochemical parameters (increased Chl and carotenoid content, relative water content, decreased relative electrolyte leakage, increased proline accumulation, and decreased malondialdehyde concentration). MnO NPs also helped plants accumulate AOEs in their leaves [177].

The combined effect of MnO NPs (13 nm) and triacontanol demonstrated a reduction in lanthanum-induced stress in tomato plants, resulting in increased antioxidant levels, reduced damage, and stimulation of growth and photosynthesis [178].

In experiments to study how ZnO_MnO NC mitigates the toxic effect of Cd contamination on soil, ZnO_MnO NC (less than 20 nm), biosynthesized from *Conocarpus erectus* L. leaf extract, was added by soil impregnation to experimental pots seeded with wheat [179]. A dose of 150 mg/L ZnO_MnO NC without Cd exposure significantly improved growth parameters (root length increased by 27.8%, shoot length by 47.9%) compared with other concentrations and compared with the control. Under Cd stress, 150 mg/L ZnO_MnO NC also resulted in a significant increase in root length and shoot length compared to the control, and increased the carotenoid and total Chl contents in plants [179]. Wang et al. [180] showed that foliar treatment of wheat with MnO_2_ NPs (rod form, average size 116 nm) at a dose of 50–100 mg/L also improved wheat growth, plant height, ear number, panicle length, antioxidant activity, and photosynthesis compared to the control under Cd stress. A 26.3% decrease in Cd concentration in wheat grain was observed. However, a dose ≥200 mg/L had a negative effect on the plant [180]. The information presented in this paragraph demonstrates the high potential of Mn_x_O_x_ NPs for stimulating physiological and biochemical processes in plants under various types of stress factors due to the modulation of antioxidant photosynthetic activity, which has a positive effect on plant productivity.

### 7.3. ZnO NPs

Research shows that Zn, as a micronutrient, is an essential plant nutrient, participating in photosynthesis, metabolic regulation, and hormone synthesis (auxins and cytokinins). Zn promotes plant resistance to stress (pathogens, drought, cold, etc.) and plays a key role in physiological processes, such as carbohydrate metabolism and maintaining cell membrane integrity, and is a major component of essential proteins [35,181].

ZnO NPs are among the most commonly synthesized MONPs and considered a highly valuable and essential material due to their multifunctional properties, stability, low cost, and wide range of applications [182].

#### 7.3.1. Stimulating Effect of ZnO NPs on Morphometric and Biochemical Parameters of Stress-Resistant Plants

The stimulating effect of ZnO NPs on the morphometric characteristics of plants, as well as their influence on some biochemical parameters, such as AOE activity, the content of LPO products, and the amount of protein and photosynthetic pigments, has been demonstrated. Thus, a positive effect of ZnO NPs (32 nm) synthesized using saponins isolated from plants on the germination and development of pearl millet (*Panicum miliaceum* L.), as well as an increase in the activity of plant protective enzymes, such as lipoxygenase, phenylalanine, polyphenol oxidase, ammonia lyase, and POD, was shown [181]. Tomato, regardless of the duration of treatment with ZnO NPs (35 nm), demonstrated a positive increase in morphometric parameters (shoot and root length, fresh and dry weight of shoots and roots, leaf area) compared to the control plants [90]. The maximum increase in tomato growth parameters, as well as AOE activity, proline accumulation and photosynthetic rate, was recorded under exposure to 8 mg/mL for 30 min [90]. ZnO NPs, which have a wurtzite structure with a particle size of 37 nm, when foliar applied at a concentration of 5 g/L, significantly improved plant height, leaf area, and biomass accumulation of pea (*Pisum sativum* L.), leading to an overall increase in yield [183].

The stimulating effect of ZnO NPs on seed treatments of various plants has been demonstrated. For example, treating maize seeds with ZnO NPs at a concentration of 8 mg/L resulted in increased root and shoot length, as well as higher protein content [91]. The application of ZnO NPs (30–50 nm) at a concentration of 2200 mg/kg to sandy fluvisol and stagnant fluvisol in humus-rich heavy loamy soils led to an increase in Zn content in plant shoots and roots [131]. ZnO NPs increased CAT activity in *H. vulgare* barley shoots grown in fluvisol [131]. ZnO NPs (7.14 nm) increased the length of rice shoots and roots across all treatments (seed treatment and foliar feeding) [184]. Seed treatment increased shoot fresh weight. Foliar feeding increased shoot fresh weight. Combined treatment with ZnO NPs increased shoot fresh weight compared to the control. Similar results were obtained for shoot dry weight and root weight. The best yield results were obtained with the combined application of treatments (seed treatment and foliar feeding). In addition, combined treatment increased Chl *a* levels [184]. The positive effect of nanopriming with ZnO NPs in combination with SiO_2_ NPs was demonstrated in the treatment of pepper (*Capsicum annuum* L.) seeds [185]. Seed germination and biometric parameters of seedlings increased, and the accumulation of proline and flavonoids was noted [185]. ZnO NPs positively affected the production of Chl pigment by maize and wheat plants. The beneficial effects of ZnO NPs on the quantitative, dietary, and physiological parameters of wheat and maize, as well as the total content of protein, carbohydrates, and fats, were studied by Singh et al. [186]. A positive effect of seed treatment with ZnO NPs was also shown in two rapeseed varieties, which stimulated shoot length, caused the accumulation of proline, soluble sugars and proteins, reduced the amount of ROS, increased AOE activity and pigment synthesis [187].

Biogenic ZnO NPs (spherical, 10–20 nm) obtained through green synthesis using duckweed *L. minor* extract at concentrations of 6 and 18 mg/L improved several growth parameters of olive tree explants *Moraiolo* cultivar, such as shoot number, green fresh mass, and total dry mass [188]. This treatment also increased the content of Chl *a* and *b*, soluble protein, carotenoids, anthocyanins, total phenolic content, and 2,2-diphenyl-1-picrylhydrazyl radical scavenging capacity. In a hydroponic experiment, foliar feeding of wild apple (*M. robusta*) plants with 200 mg/L ZnO NPs (30 nm) was shown to have a positive effect on the biometric characteristics of plants (enhanced growth, increasing height, total dry mass of plants, and root length). At the biochemical level, this treatment improved nutrient absorption, reducing oxidative stress [93].

Peanut seeds treated with ZnO NPs (25 nm, 1000 ppm) showed improved germination, seedling vigor, plant growth, Chl content, and earlier flowering compared with bulk ZnSO_4_ [189]. ZnO NPs also enhanced root and stem growth and increased pod yield by 34%. Field experiments confirmed higher yields with foliar ZnO NPs applied at much lower doses than ZnSO_4_, while excessive concentrations (2000 ppm) inhibited growth [189].

The mechanism for increasing Chl content is based on the fact that Zn is an essential plant nutrient and is involved in plant metabolism by modifying the action of vital enzymes [59]. Foliar application of ZnO NPs (10 ppm) to 14-day-old clusterbean (*Cyamopsis tetragonoloba* L.) plants significantly improved growth and physiological parameters after six weeks [190]. Plant biomass, shoot and root growth, Chl content, soluble leaf protein, rhizospheric microbial population, and enzyme activities (acid and alkaline phosphatase, phytase) all increased compared with the control. In addition, seed gum content rose by 7.5%, indicating the potential of ZnO NPs to enhance both agricultural productivity and industrial value [190]. The increase in photosynthetic properties after exposure to ZnO NPs may be due to increased light absorption, which further protects chloroplasts from aging and prolongs the photosynthetic period of chloroplasts, ultimately leading to increased photosynthesis [191,192,193]. The results suggest that the use of ZnO NPs increases crop yield, with this effect observed with increasing concentrations and with simultaneous application of NPs to both seeds and foliar feeding [194].

The ZnO_MnO complex with particle sizes of 9.5 and 10.5 nm enriched with sugarcane bagasse ash and Andean blueberry (*Vaccinium myrtillus* L.) extract as a foliar fertilizer for cabbage (*Brassica oleracea* var. *capitata*) at concentrations of 270 and 540 mg/L resulted in an increase in root size, leaf area, dry biomass, and Chl content compared to the control sample [195].

#### 7.3.2. The Effect of ZnO NPs on Plant Stress Resistance

Several studies have been conducted on enhancing plant resistance to phytopathogens using ZnO NPs. Tomato infected with the soil-borne bacterial plant pathogen *R. solanacearum* were grown in soil treated with spherical ZnO NPs (16–31 nm) at a concentration of 500 mg/kg [151]. The use of ZnO NPs resulted in an increase in tomato plant length, fresh weight, and dry weight. The treatment reduced MDA content and increased POD and SOD content in tomato plants compared to the control. Thus, ZnO NPs significantly improved the growth of tomato infected with brown spot, mitigating the effects of the disease compared to the control [151].

Spraying potato plants with ZnO NPs at a concentration of 100 mg/L in a greenhouse experiment reduced *P. infestans* infestation by 70.64%, while spraying at a concentration of 50 mg/L reduced the infestation by 47% compared to the control [154]. ZnO NPs synthesized using *Penicillium expansum* in vivo suppressed *Fusarium* wilt of eggplant *Solanum melongena* L., reducing disease severity by 75% and providing plant protection against *Fusarium oxysporum* [161]. Increases in biometric parameters, photosynthetic pigment content, proteins, phenolics, carbohydrates, and AOE activity were observed [161].

ZnO NPs can enhance plant resistance to abiotic stress. Thus, foliar feeding of lettuce with ZnO NPs (diamond-shaped, 15–35 nm) grown in soil contaminated with phenanthrene (Phe, 50 mg/kg) and Cd (5.36 mg/kg) increased fresh biomass by 27.2% (at a low ZnO NP concentration of 20 mg/L) and by 8.42% (at a high ZnO NP concentration of 100 mg/L) [196]. The root length of lettuce increased by 20.4% and 39.6% at a low and high dose of ZnO NPs, respectively. Treatment with Phe + Cd + ZnO NPs (low dose) and Phe + Cd + ZnO NPs (high dose) enhanced the plant cell defense system against excessive solar radiation by 1.63% and 9.90%, respectively, thereby maintaining normal photosynthetic function in lettuce leaves. Foliar application of ZnO NPs decreased MDA and H_2_O_2_ levels under treatment with Phe + Cd + ZnO NPs (low dose) and Phe + Cd + ZnO NPs (high dose), respectively, which reduced the oxidative damage induced by Phe + Cd stress, especially when using a low dose of ZnO NPs [196]. Another study also showed that ZnO NPs enhanced the tolerance of maize plants to Cd stress [197]. ZnO NPs increased proline content and AOE activity (SOD, POD, CAT, and APX) [197]. Similar stimulatory effects of ZnO NPs on maize plants under Cd stress were shown also in [198].

There are a number of works devoted to plant resistance to salt stress under the influence of ZnO NP. For instance, foliar treatment of annual vegetable plant okra (*Abelmoschus esculentus* L. Moench cv. Hasawi) with ZnO NPs at a concentration of 10 mg/L under salt stress conditions with seawater at concentrations of 0, 10, 25, 50, 75, and 100% SW increased the total amount of proline and soluble sugars and stimulated AOE activity [199]. ZnO NPs exerted a prolonged effect on the adaptation of alfalfa (*M. sativa*) callus to NaCl-induced salt stress. ZnO NPs regulated the functioning of the antioxidant system (reduced the MDA level in plant tissues, regulated the formation of H_2_O_2_), which contributed to optimal protein synthesis [163]. Foliar feeding of radish (*R. sativus*) with ZnO NPs under salt stress resulted in a decrease in oxidative stress, an increase in leaf area, and an improvement in a number of parameters of photosynthetic activity and mineral content in radish tissues [200]. The addition of ZnO NPs increased the content of photosynthetic pigments, increased the activity of AOEs, and decreased the accumulation of proline and total soluble sugars in okra compared to the control [199].

In Bashir et al. [32], the combination of ZnO NPs and co-composted biochar (0.5% *w*/*w*) improved wheat growth and biomass increase, Chl content, and AOE activity by scavenging ROS and reducing plant Cd uptake under simultaneous Cd and drought, salt stress conditions.

Under salt stress, which reduced all growth parameters in peas, ZnO NPs added at a concentration of up to 50 mg/L increased root length and improved other physiological parameters compared to control samples [201]. A decrease in MDA, glycine betaine, and hydrogen peroxide levels was also observed [201]. Treatment of rice plants exposed to salt stress with ZnO NPs (<100 nm) restored their growth and productivity to levels comparable to those of unstressed control plants [202]. The following growth parameters improved: plant height, root length, shoot number, and fresh produce weight [202]. Improved growth parameters, photosynthetic efficiency, and antioxidant system function were also observed in pumpkin (*Cucurbita pepo* L.) grown under water-stress conditions on saline soil after treatment with ZnO NPs [203]. Exogenous application of spheroidal ZnO NPs with an average size of 30 nm and a length of 0.2 μm under salt stress significantly increased morphological parameters of rice, such as shoot length, root length, shoot weight, and root weight [204]. A 32% increase in Chl content and a 22% increase in all photosynthesis parameters were observed compared to plants treated with salt alone. In stressed rice, ZnO NPs reduced H_2_O_2_ and MDA accumulation by 14% and 19%, respectively, compared to plants treated with water alone [204].

Thus, the protective effect of ZnO NPs on plants is supported by a large number of published studies. These studies confirm the potential of ZnO NPs as eustressors, helping to enhance plant resilience to a variety of stress factors.

**Figure 4:**
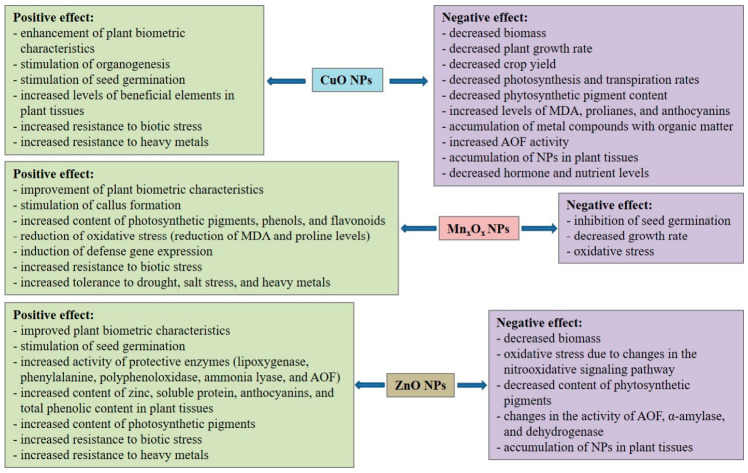
Brief chart of the negative and positive biological and eustress effects of metal oxide nanoparticles (MONPs), CuO, Mn_x_O_x_, ZnO NPs, on plant growth and development.

Brief chart of the negative and positive biological and eustress effects of MONPs (CuO, Mn_x_O_x_, and ZnO NPs) on plant growth and development is presented in Figure 4.

A comparison of various methods of applying MONPs to plants (foliar application, spraying, and seed nanopriming) shows that the results largely depend on the NPs used and the plant species. Nanopriming using MONPs acts as a protective coating for seeds, reducing susceptibility to seed-borne diseases. MONPs improve seed germination and sprouting, promote water absorption, and increase enzymatic activity in seeds. However, due to the different characteristics of seeds, not every plant can be subjected to seed nanopriming. It is important to consider the characteristics of the NPs, select the optimal concentration, and select the application interval to avoid negative effects from nanopriming. MONPs have great potential when applied to plants by spraying. In particular, applying MONPs to plant leaves helps protect them from UV radiation. This helps reduce damage caused by excessive solar radiation, which can lead to decreased photosynthesis and reduced yields. The best yield results are achieved with a combination of treatments (for example, seed treatment and foliar feeding). However, when planning any MONP treatment, it is necessary to consider the plant species, the length of its growing season, the soil characteristics, and the growing climate, and select the optimal MONP concentrations.

## 8. Optimizing the Use of NPs to Reduce Toxicity and Increase Their Efficiency

The harmful effects of MONPs on plants can be reduced through a well-designed program for the creation and application of nanostructures. The degree of NP toxicity depends on land use, soil properties, the amount of organic matter, pH, cation exchange capacity, and redox potential [205]. Untreated and infertile soils have been shown to be more susceptible to the toxic effects of NPs than treated soils [206]. Accordingly, soils richer in mineral and organic matter not only play an important role for plants, microorganisms, and invertebrates (serving as a primary source of water and nutrients, providing habitat, etc.) but also facilitate the modification of the NP surface, thereby reducing surface reactivity and ion release, which reduces toxicity, especially at high concentrations [207]. High organic matter content binds NPs, reducing their bioavailability and preventing their direct interaction with soil organisms [208]. Modification of the NP surface with polymers, surfactants, humic acids, and proteins can improve the dispersion of NPs in an aqueous environment, preventing their agglomeration and ensuring a more uniform distribution in soil and plant tissues [209]. Chemical modification (with polyethylene glycol, polyvinylpyrrolidone) of the NP surface changes the charge density and hydrophobicity, reduces the toxicity of NPs and increases their efficiency [210,211].

Stabilization of NPs with polysaccharides (arabinogalactan, starch, cellulose, alginate, chitin, and chitosan) reduces the toxicity of NPs to plants by increasing the aggregative stability of particles and changing their physicochemical properties, which affects their interaction with biological systems [212,213,214]. The influence of polysaccharides on the physicochemical properties of NPs is possible due to the following key mechanisms. First, steric stabilization, which is a critical stabilization mechanism for disperse systems (emulsions, foams, suspensions) against coagulation and coalescence: It is a “non-thermodynamical” factor formed by adsorption layers of surfactants or polymers that form quasi-two-dimensional, solid-like, elastic structures at the interfaces. Long-chain polysaccharide molecules are adsorbed on the surface of NPs, forming a dense protective hydrated layer on their surface, thereby preventing the particles from approaching each other and reducing the likelihood of aggregation. Second, electrostatic stabilization: highly polar functional groups of polysaccharide macromolecules (e.g., hydroxyl and terminal carbonyl groups) can interact with the surface of NPs, providing electrostatic repulsion between the NPs. This increases their aggregative stability. However, the ability of charged macromolecules to stabilize NPs depends on the number of charged units in the polymer chain, as well as the pH of the medium and the presence of low-molecular-weight electrolytes. Third, controlling NP size during synthesis: Polysaccharides can influence the diffusion of precursor molecules to the surface of growing NPs, allowing for the regulation of their size. Fourth, forming hybrid systems: polysaccharides can act as a matrix in which NPs are uniformly distributed. This facilitates the formation of a stable, water-soluble system. For example, NPs can be dispersed in a polysaccharide matrix, which improves their stability and expands their application range. And fifth, polysaccharides protect NPs from external influences. NPs stabilized on polysaccharide matrices may have a longer shelf life. When the biopolymer dries, a strong film forms that protects the NPs from external influences [215,216]. Furthermore, polysaccharide matrices allow the creation of composites with controlled, slow-release NPs with an extended duration of action, effectively preventing toxic effects during rapid delivery of large doses [214,217]. NP coatings can provide biocompatibility and increase stability [218]. These surface modifications are used to enhance the beneficial effects or reduce the potential toxicity of NPs. Thus, NP surface modification allows for greater control over the physical and chemical properties of NPs. In sandy soils and soils with low organic matter content, NPs remain more bioavailable and mobile, increasing their contact with biological surfaces (e.g., microbial cells) [219]. Thus, the toxicity of NPs depends on the characteristics and properties of their surface, as well as their chemical composition.

Another important soil characteristic when assessing the toxicity of NPs is its pH, which affects the dissolution and release of ions, which consequently influences the mechanisms of toxicity. Acidic soils can increase the solubility and release of ions from metal-based NPs, making them more bioavailable and therefore more likely to be taken up by plants and microorganisms, enhancing their toxic effects [219,220]. Meanwhile, soil alkalinity promotes the aggregation of NPs, causing them to lose their nanospecific properties and the rate of subsequent dissolution into ions to decrease. Alkaline pH-induced aggregation reduces the retention and transport of NPs in the soil and, accordingly, leads to a weakening of their effects on biological systems [221,222].

Other known factors such as NP size, dose, and contact time can also significantly affect the effectiveness of NPs. NP size is one of the factors determining the transport, fate, and bioavailability of NPs in agricultural environments, as well as their interactions with soil particles, water, plants, and microorganisms. Smaller NPs have greater mobility in the soil environment due to a lower gravitational sedimentation rate, lower aggregation potential, and more active Brownian motion, which facilitates their penetration through soil pores and root tissues [223,224]. In contrast, large NPs are often immobilized as a result of strong interactions with soil colloids and organic macromolecules, which limits their mobility and environmental impact [13]. Furthermore, NPs with positive zeta potential are more prone to toxicity than NPs with negative zeta potential due to the fact that NPs have stronger electrostatic interactions with negatively charged cell membranes, thereby increasing cellular uptake of NPs, leading to higher cellular levels [211,225,226]. Furthermore, the size of the NPs influences the rate and extent of their dissolution, especially in the case of MONPs. The smaller the NP size, the greater their surface curvature and free energy, which leads to increased dissolution rates and ion release—factors that increase toxicity to soil microorganisms and plants [227]. NPs with diameters ranging from 10 to 40 nm have been shown to optimally penetrate plant tissue [228].

The studies reviewed demonstrated that the effects of CuO, ZnO, and Mn_x_O_x_ NPs on plants are related to their concentration. For example, CuO NPs at concentrations of 50 to 200 mg/mL can exert a positive stimulating effect on plants, but with concentrations above 200 mg/mL, a negative effect is observed. The toxicity of ZnO and Mn_x_O_x_ NPs to plants was dose-dependent, also known as a hormesis effect: low doses (up to 50 mg/mL) promoted stimulation, while high doses (>100 mg/mL) gradually aggravated the inhibitory effect.

Another key factor influencing NP interactions with plants, tissue penetration, and intraplant transport is the NP surface charge. This determines how NPs interact with cellular structures, biochemical processes, and other plant components. Modifying the NP surface can alter its charge, which is often used to improve the efficiency of NP uptake and targeted delivery [229]. Studies have shown that positively charged NPs adhere more strongly to plant surfaces than negatively charged ones due to electrostatic attraction to the negatively charged cell wall. Unlike adhesion, positively charged NPs penetrate plant tissue in smaller quantities compared to negatively charged ones. Herbaceous plants most readily absorb positively charged NPs through their roots, while negatively charged ones are most effectively transported from roots to shoots (including stems and leaves) [230,231]. Therefore, the appropriate method of NP application (roots, leaves), taking into account their charge, may enable the desired positive effect on plants.

In addition to various abiotic factors, certain biotic factors can also influence NP toxicity. For example, arbuscular mycorrhizal fungi (AMF) may play a positive role in reducing the negative impacts and environmental risks associated with NPs in agroecosystems [19]. This is attributed to the ability of AMF to release glycoprotein glomalin, which can act as a binder, aggregating soil mineral particles and preventing nutrient leaching, which may indirectly reduce the toxic effects of NPs by improving the conditions for the life of soil microorganisms and plants [232,233]. It has also been shown that exposure to ZnO NPs caused an increase in the production of siderophores (small, high-affinity iron-chelating compounds secreted by microorganisms) in plant root-associated bacteria, such as *Pseudomonas chlororaphis*. Siderophores secreted by bacteria have been shown to reduce metal uptake by plants [234]. In rhizosphere soil, plant root exudates, such as citric acid, can also determine the aggregation, sedimentation, and dissolution of CuO NPs [235]. Given the inherent differences among various synthesized NPs, it is important to develop and utilize NPs or nanomaterials with well-controlled physical (e.g., size) and chemical (e.g., surface coatings) properties to facilitate research aimed at elucidating the relationships between the properties of nanomaterials and their environmental impacts.

## 9. Conclusions

Thus, this review of published data demonstrates that MONP characteristics (size, surface charge, and coverage) and soil properties (pH, organic matter content) modulate their behavior, either enhancing or reducing their impact on plant and soil communities (plants, invertebrates, bacteria). Designing MONPs with tailored properties (size, shape, and surface characteristics) for specific soils (taking into account soil properties) leads to maximum benefits while minimizing environmental risks. The obtained data indicate that the type, concentration, form and other physicochemical and biological characteristics of NPs should be taken into account when assessing their overall fate, transport routes, and impacts on plants and the environment. MONPs (CuO, Mn_x_O_x_, and ZnO NPs), when used correctly, improve the morphological and physiological–biochemical characteristics of plants and can be used as micronutrients, stimulating plant growth and thereby increasing yields. The studied MONPs promote plant growth and development, significantly enhance disease resistance, and mitigate stress factors. MONPs are effective due to their small size and ability to deliver essential substances. These properties contribute to positive changes in plant morphology, anatomy, and physiology. MONPs can act as eustressors within a certain concentration range. Doses of MONPs above threshold concentrations can cause phytotoxicity, primarily due to the induction of oxidative stress. An inhibitory effect of high concentrations on plant growth has been observed. These properties of MONPs indicate the need for additional research to elucidate the molecular mechanisms of MONP-mediated stress responses under various conditions. In the future, it is necessary to develop effective and safe formulations for using MONPs as eustressors, taking into account the characteristics of each plant species, application methods, and concentrations. This will facilitate the development of methods for their effective use in agriculture, contributing to food security.

## Figures and Tables

**Table 1:** Dose-dependent biological effects of metal oxide nanoparticles (MONPs) on plants.

Plant	Application Method	Effect	Concentration		Reference
			Negative	Safe	
**CuO NPs**					
Willow (*Salix fragilis* L.)	soil	plant growth rate	500 mg/kg	100 mg/kg	[71]
Sweet cherry (*Prunus avium* L.)	seeds	plant growth rate	400–1000 mg/L	200 mg/L	[72]
Mustard (*Brassica nigra* (L.) W.D.J. Koch)	seeds	seed germination, plasma membrane integrity, and potassium ion (K^+^) leakage rate	1000 mg/L	250, 300 mg/L	[73]
	leaves	AOE activity and the intensity of photosynthesis processes	16 mg/L	8 mg/L	[74]
Cucumber (*Cucumis sativus* L.)	soil	intensity of plant growth, accumulation of NPs and nutrients in plant tissues	1000 mg/kg	100, 200 mg/kg	[75]
Weed and cultivated rice	soil	crop formation, plant hormonal status, accumulation of microelements	≥300 mg/kg	75, 150 mg/kg	[76]
Rice (*Oryza sativa* L.)	hydroponics	plant growth rate and AOE activity, development of oxidative stress	100 mg/L	50 mg/L	[77]
Lettuce (*Lactuca sativa* L.)	roots	root growth rate	450 mg/L	5 mg/L	[78]
**Mn_x_O_x_ NPs**					
Lettuce (*L. sativa*)	seeds	seed germination	no data	0.025–10 mg/L	[79]
Watermelon (*Citrullus lanatus* (Thunb.) Matsum & Nakai)	seeds	seed germination, hormonal status, content of photosynthetic pigments, development of oxidative stress	40, 80 mg/L	10–20 mg/L	[80]
Belladonna (*Atropa belladonna* L.)	agar-supported Murashige–Skoog medium	AOE activity, development of oxidative stress	50–200 mg/L	25 mg/L	[81]
Radish (*Raphanus sativus* L.)	soil	development of oxidative stress and the absorption of essential metallic mineral elements by the plant	100 mg/L	10–50 mg/L	[83]
Tomato (*Solanum lycopersicum* L.)	leaves	AOE activity, development of oxidative stress	50 mg/L	10 mg/L	[82]
**ZnO NPs**					
Rapeseed (*Brassica napus* L.)	seeds	root growth rate	>10 mg/L	0.1–10 mg/L	[84]
Mustard (*B. nigra*), rapeseed (*B. napus*)	seeds	plant growth rate, AOE activity, development of oxidative stress	100 mg/L	25 mg/L	[85]
Wheat (*Triticum aestivum* L.)	seeds	plant growth rate	125, 250, 500 mg/L	62 mg/L	[86]
	hydroponics	root growth rate and chlorophyll content	2–4 mg/L	1 mg/L	[87]
Tomato (*S. lycopersicum*)		growth intensity and photosynthetic activity, AOE activity	400, 800 mg/L	200 mg/L	[90]
	soil	AOE activity, content of protein, proline and photosynthetic pigments	100, 200 mg/L	10 mg/L	[59]
*Abelmoschus esculentus* L. Moench	seed priming and foliar spraying	growth rate	50 to 200 mg/L	20 mg/L	[88]
Maize (*Zea mays* L.)	seeds	growth rate and protein content	no data	>8 mg/L	[91]
Coffee (*Coffea arabica* L.)	soil	development of oxidative stress, intensity of photosynthesis in conditions of soil acidification	50–100 mg/L	10–25 mg/L	[92]
	leaves	AOE activity, development of oxidative stress, amount of photosynthetic pigments under salinity conditions	100 mg/L	50 mg/L	[70]
Wild apple (*Malus robusta*)	hydroponics	effect on biometric characteristics and components of the plant antioxidant system	500–1000 mg/L	200 mg/L	[93]

## Data Availability

The data presented in this review are all publicly available in the published papers referred to in this review.

## Abbreviations

The following abbreviations are used in this manuscript:

AOEs	antioxidant enzymes
APX	ascorbate peroxidase
Ca	calcium
CAT	catalase
Cd	cadmium
Chl	chlorophyll
Cu	copper
Fe	iron
GPX	glutathione peroxidase
H_2_O_2_	hydrogen peroxide
K	potassium
LPO	lipid peroxidation
MDA	malondialdehyde
MD	maltodextrin
Mg	magnesium
MONPs	metal oxide nanoparticles
Na	sodium
NC	nanocomposite
NMs	nanomaterials
Mn	manganese
NPs	nanoparticles
Phe	phenylalanine
POD	peroxidase
RNA	ribonucleic acid
ROS	reactive oxygen species
SOD	superoxide dismutase
SR	systemic resistance
XTH	xyloglucan endotransglycosylase/hydrolase
Zn	zinc
